# A new species of Themus (Themus) Motschulsky from Yunnan, China and a redescription of T. (T.) testaceicollis Wittmer, 1983 (Coleoptera, Cantharidae)

**DOI:** 10.3897/zookeys.525.6021

**Published:** 2015-10-05

**Authors:** Junyan Su, Limei Li, Yuxia Yang, Xingke Yang

**Affiliations:** 1 The Key Laboratory of Zoological Systematics and Application, College of Life Sciences, Hebei University, Baoding 071002, Hebei Province, China; 2 Key Laboratory of Zoological Systematics and Evolution, Institute of Zoology, Chinese Academy of Sciences, Beijing 100101, China

**Keywords:** Taxonomy, Cantharidae, *Themus*, new species, China

## Abstract

A new species is described, Themus (Themus) dimorphus **sp. n.** from Yunnan, China. Themus (Themus) testaceicollis Wittmer, 1983 is redescribed and compared with the new species. The two species are illustrated with habitus and genitalia of both sexes and abdominal sternites VIII of female.

## Introduction

The genus *Themus* Motschulsky, 1858 was divided into four subgenera (Wittmer 1973, 1997), whose diagnoses were redefined by Švihla (2008). The nominotypical subgenus includes more than 100 species widely distributed in the Oriental and eastern Palaearctic regions (Švihla 2008). In China, approximately 50 species of this subgenus are known, and most species were described or revised by Wittmer (1983).

In recent study, a new species was discovered from Yunnan, China and is described here under the name of Themus (Themus) dimorphus sp. n. It is similar to Themus (Themus) testaceicollis Wittmer, 1983, which is redescribed in the present study to make comparisons with the new species.

## Material and methods

The types or other specimens are deposited in the following collections:

IZAS Institute of Zoology, Chinese Academy of Sciences, Beijing, China;

MHBU Museum of Hebei University, Baoding, China;

MNHN Muséum national d’Histoire naturelle, Paris, France;

NHMB Naturhistorisches Museum Basel, Switzerland.

The studied method and description format follow that of Yang et al. (2014). Morphological terminology of female genitalia follows that of Brancucci (1980) and abbreviations in the figures are listed as follows: **ag** accessory gland; co coxite; di diverticulum; tg9 abdominal tergite IX; sp spermatheca; ov median oviduct; va vagina; sr sclerotized bracket or ridge.

## Taxonomy

### Themus (Themus) dimorphus

Taxon classificationAnimaliaColeopteraCantharidae

Y. Yang & X. Yang sp. n.

**Figure 1.**
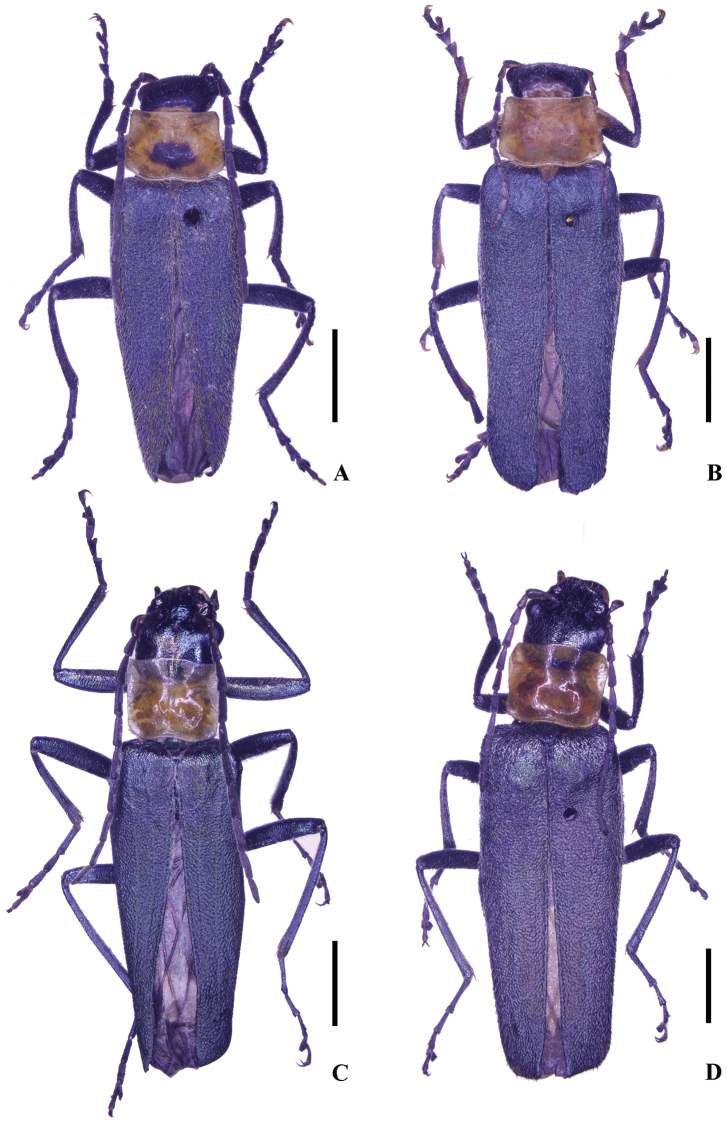
Habitus, dorsal view (**A, C** male **B, D** female): **A–B** Themus (Themus) dimorphus sp. n. **C–D** Themus (Themus) testaceicollis Wittmer, 1983. Scale bars = 2.0 mm.

**Figure 2.**
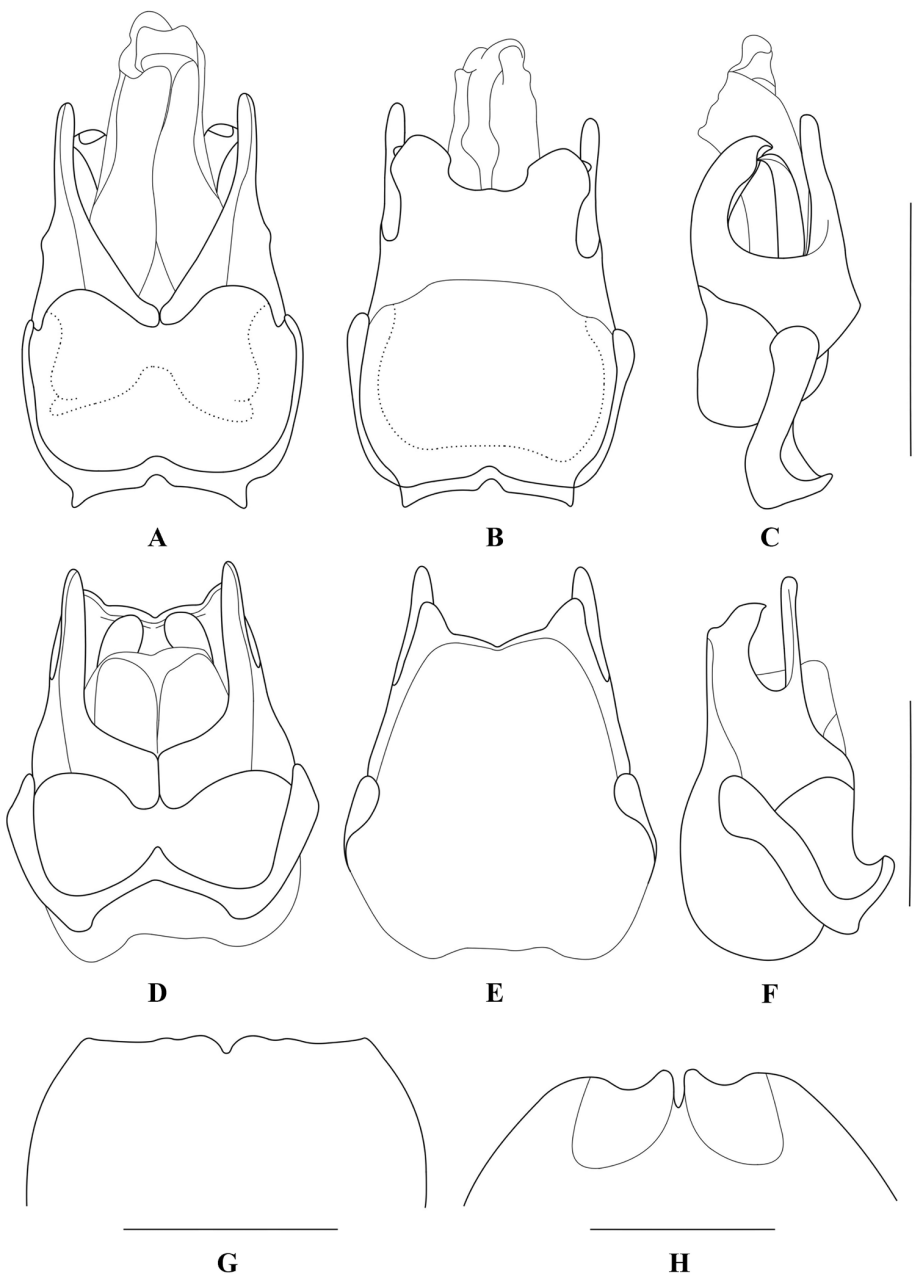
**A–F** Aedeagus (**A, D** ventral view **B, E** dorsal view **C, F** lateral view); G–H. abdominal sternite VIII of female, ventral view: **A–C, G** Themus (Themus) dimorphus sp. n. **D–F, H** Themus (Themus) testaceicollis Wittmer, 1983. Scale bars = 1.0 mm.

**Figure 3.**
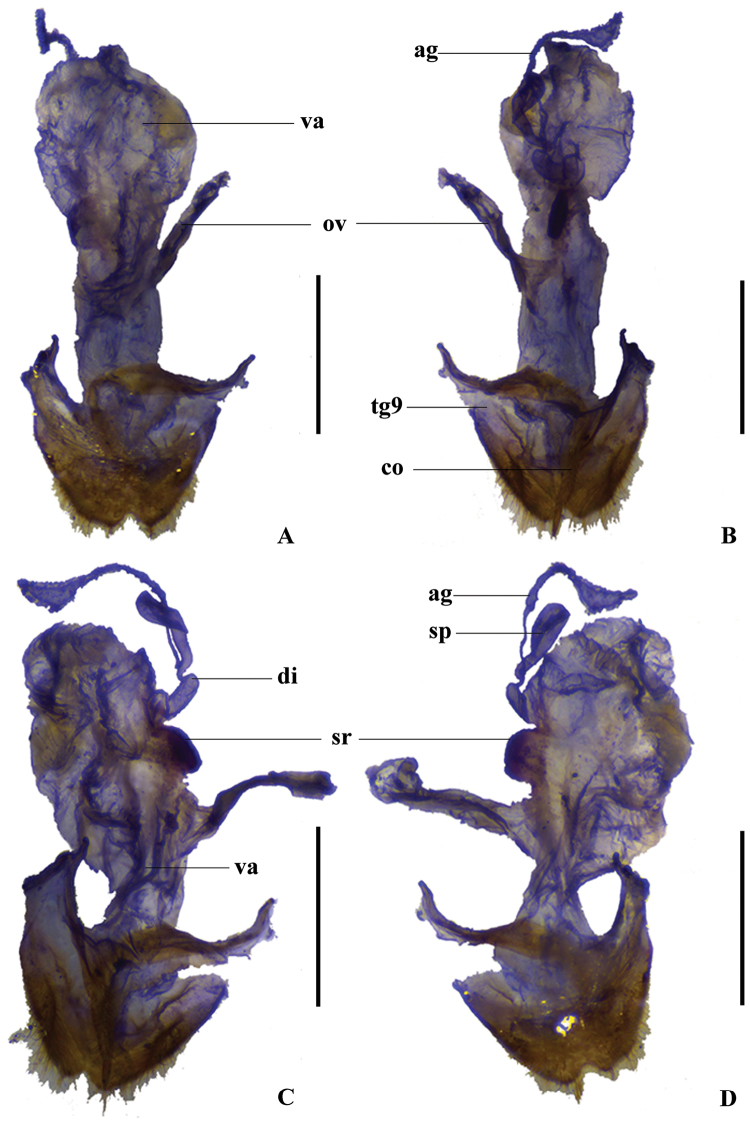
Female genitalia of Themus (Themus) dimorphus sp. n.: **A** dorsal view **B** ventral view **C** right lateral view **D** left lateral view. Scale bars = 1.0 mm.

Figs 1A–B , 2A–C, G , 3

#### Type material.

Holotype, male (MHBU): **CHINA**: Yunnan, Yiliang, Xiaocaoba, 25.VIII.2013, leg. Xun Bian & Guang-Lin Xie; Paratypes: 2 males (MHBU): same data to the holotype; 2 females (MHBU): same locality and collectors, 24.VIII.2013.

#### Description.

Male (Fig. 1A). Head metallic dark blue and weakly shining, lateral parts of clypeus yellow, mouthparts yellow, maxillary and labial palpi and apices of mandibles dark brown; prothorax yellow, pronotum with a moderately large, irregular black marking in center of disc, antennae black, antennomeres I–III yellow on ventral sides, scutellum black, elytra metallic green and weakly shining, legs and meso- and metasterna metallic dark blue and weakly shining, abdomen yellow, slightly darkened on both sides of ventrites I–V. Body densely covered with decumbent brown pubescence, mixed with slightly long semierect pubescence on elytra and a few long hairs along anterior margin of clypeus.

Head rounded, densely and finely punctate, surface lustrous; eyes slightly protruding, breadth across eyes slightly wider than anterior margin of pronotum; terminal maxillary palpomeres widest nearly in middle, arcuate and sharp at apical parts of inner margins, rounded at apices; antennae extending to basal two-fifth length of elytra, antennomeres II approximately twice as long as wide at apices, III–X slightly obliquely widened apicad, III approximately twice as long as wide at apices and 1.5 times as long as II, IV approximately 1.5 times as long as III, IV–XI subequal in length, each with a small round to short narrow longitudinal impression in middle or basal part of outer margin, XI pointed at apices.

Pronotum approximately 1.40 times as wide as long, widest near base, anterior margin arcuate, lateral margins slightly sinuate at anterior part and distinctly diverging posteriorly, posterior margin bisinuate and narrowly bordered, anterior angles rounded, posterior angles nearly rectangular, disc slightly convex at posterolateral parts, surface lustrous, slightly finely and sparsely punctate than that on head.

Elytra approximately 3.0 times longer than pronotum, 2.6 times longer than humeral width, lateral margins distinctly converging posteriorly, apex of each elytron rounded, disc rugulose-lacunose and coarsely punctate, surface matt.

Aedeagus (Fig. 2A–C): ventral process of each paramere narrow and straight, rounded at apex; conjoint dorsal plate of parameres slightly shorter than ventral processes, largely subroundly emarginated in middle of apical margin, with lateroapical angles acute and bent ventrally; laterophyses widely separated on both sides of median lobe, with apices acute and pointed dorsally to lateroapical angles of conjoint dorsal plate.

Female (Fig. 1B). Similar to male, but clypeus uniformly yellow; antennae narrower and shorter, extending to basal one-fifth length of elytra; antennomeres III nearly as long as II, IV‒XI without impressions, XI slightly longer than X; pronotum without any black marking (one specimens with two indistinct small dark brown markings on both sides of disc); elytra approximately 2.5 times longer than humeral width, with lateral margins slightly converging posteriorly; scutellum sometimes yellow (one specimen). Abdominal sternite VIII (Fig. 2G) moderately triangularly emarginated in middle and indistinctly emarginated on both sides of posterior margin. Internal reproductive organ of genitalia (Fig. 3): vagina stout, abruptly narrowed into a short tube at apical part of ventral side, below which present with a pair of strongly sclerotized, arcuate and conjoint ridges extending to median oviduct; diverticulum and spermatheca arising from the end of the short tube of vagina; diverticulum very short and rounded at apex; spermatheca sac-shaped and rounded at apex, distinctly expanded apicad and bent in middle, provided with a moderately long and thin accessory gland at base.

Body length: 8.0‒11.0 mm; width: 3.0‒4.5 mm.

#### Distribution.

China (Yunnan).

#### Etymology.

The specific name is derived from Latin *dimorphus* (dimorphic), referring to its different coloration of head and pronotum in both sexes.

#### Diagnosis.

The new species can be distinguished from most of the species of Themus (Themus) Motschulsky by the smaller body and uniformly orange pronotum or at most with one or two small black markings, which characters also equipped by the following species in the key.

#### A key to Themus (Themus) dimorphus sp. n. and its similar species

**Table:** 

1	Legs mixed with orange and blue	**2**
–	Legs uniformly blue	**3**
2	Pronotum distinctly wider than long, with lateral margins nearly parallel; aedeagus: laterophyses flattened and tapered apically, slightly longer than conjoint parameres of parameres	**Themus (Themus) tumlonganus (Pic, 1916)**
–	Pronotum slightly wider than long, with lateral margins slightly converging posteriorly; aedeagus: laterophyses unlike above, stout, slightly shorter than conjoint dorsal plate of parameres	**Themus (Themus) foveicollis (Fairmaire, 1900)**
3	Pronotum distinctly wider than long, with lateral margins slightly diverging posteriorly; aedeagus: conjoint dorsal plate of parameres subroundly emarginated in middle of apical margin	**Themus (Themus) dimorphus sp. n.**
–	Pronotum slightly wider than long, with lateral margins slightly converging posteriorly; aedeagus: conjoint dorsal plate of parameres inverse-trapeziformly emarginated in middle of apical margin	**Themus (Themus) testaceicollis Wittmer, 1983**

### Themus (Themus) testaceicollis

Taxon classificationAnimaliaColeopteraCantharidae

Wittmer, 1983

**Figure 4.**
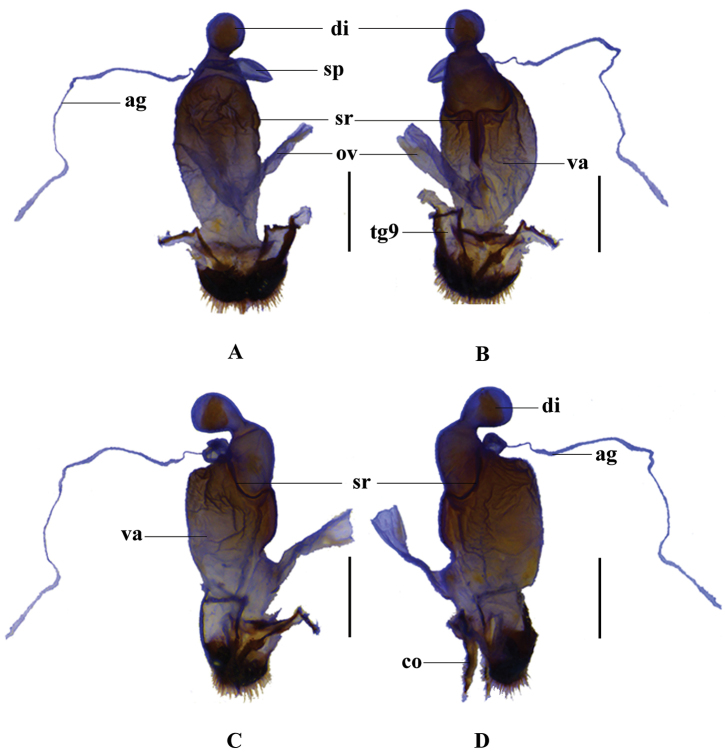
Female genitalia of Themus (Themus) testaceicollis Wittmer, 1983: **A** dorsal view **B** ventral view **C** right lateral view **D** left lateral view. Scale bars = 1.0 mm.

Figs 1C–D , 2D–F, H , 4

Themus (s. str.?) testaceicollis Wittmer, 1983: 218, Fig. 97.
Themus (s. str.) testaceicollis : Wittmer 1995: 127.

#### Type material examined.

Holotype: 1 female (MNHN): [h] “Tatsien Lu \ 1896” [CHINA, Sichuan, Kangding], [h] “Themus (s. str.?) \ testaceicollis \ Wittm. \ det. W. Wittmer”, [p] “HOLOTYPUS”. Paratype: 1 female (NHMB): [p] “Ta-tsien-Loû \ Chasseurs Thibétaíns \ 1896” [CHINA, Sichuan, Kangding], [h] “Themus (s.str.?) \ testaceicollis \ Wittm. \ det. W. Wittmer”, [p] “PARATYPUS”.

#### Additional material examined.

**CHINA: Shaanxi**: 12 males, 7 females (IZAS): Taibai, Baiyuanxiang, Primary Forest, 33.814°N, 107.618°E, 1619 m, 19.VI.2012, leg. Sha Li; 4 males (IZAS): Liuba, Shangtaizi, Zibaishan, 22.VI.2012, leg. Yi Hua; 1female (MHUB): Fengxian, Heigou, 13.VI.2005, leg. Yi-Bin Ba; **Hubei**: 1 female (MHUB): Badong, Lvcongpo, 1700 m, 16.VII.2006, leg. Li Cai; **Sichuan**: 1 female (MHUB): Jiuzhaigou, Xinyangerlin, 14.VII.2009, leg. Zhen-Hua Gao & Yi-Ping Niu.

#### Redescription.

Male (Fig. 1C). Head metallic green and strongly shining, mouthparts metallic green, mandibles light yellow at bases and dark brown at apices, terminal maxillary and labial palpomeres dark brown, antennae black, antennomeres I‒II metallic green on dorsal sides, I‒VI or VIII orange on ventral sides, prothorax light yellow, pronoum orange in center of disc, scutellum, elytra and legs metallic green and strongly shinning, meso- and metasterna and abdomen metallic dark blue and strongly shining. Head and elytra densely covered with decumbent light brown pubescence, pronotum with black pubescence, meso- and metasterna and abdomen with gray pubescence, sparsely mixed with long erect pubescence along anterior margin of clypeus.

Head rounded, densely and finely punctate, surface lustrous; eyes slightly protruding, breadth across eyes slightly narrower than anterior margin of pronotum; terminal maxillary palpomeres widest nearly in middle, arcuate and sharp at apical half parts of inner margins, rounded at apices; antennae almost extending to mid-length of elytra, antennomeres II approximately twice as long as wide at apices, III‒X slightly obliquely widened apicad, III about 1.5 times as long as wide at apices and slightly longer than II, IV approximately 1.5 times longer than III, IV‒VIII subequal in length and slightly longer than the following antennomeres, IV‒XI each with a short longitudinal impression nearly in middle of outer margin, XI slightly longer than X and pointed at apices.

Pronotum approximately 1.20 times as wide as long, widest before middle, anterior margin arcuate, lateral margins slightly sinuate and converging posteriorly, posterior margin bisinuate and narrowly bordered, anterior angles nearly rectangular, posterior angles rounded, disc slightly convex at posterolateral parts, finely and sparsely punctate, surface lustrous.

Elytra approximately 3.5 times longer than pronotum, 2.7 times longer than humeral width, lateral margins distinctly converging posteriorly, apex of each elytron rounded, disc rugulose-lacunose and coarsely punctate, surface matt.

Aedeagus (Fig. 2D–F): ventral process of each paramere narrow and nearly straight, rounded at apex; conjoint dorsal plate of parameres distinctly shorter than ventral processes, largely inverse-trapeziformly emarginated in middle of apical margin, present with two narrow transverse ridges near bottom of middle emargination on inner surface, lateroapical angles acute and bent ventrally in lateral view; laterophyses separated on dorsal side of median lobe, slightly shorter than conjoint dorsal plate, acute at apices which pointed towards the lower transverse ridge of conjoint dorsal plate.

Female (Fig. 1D). Similar to male, but eyes less protruding; antennae narrower and shorter, extending to basal one-fourth length of elytra; pronotum approximately 1.25 times as wide as long; elytra with lateral margins nearly parallel. Abdominal sternite VIII (Fig. 2H) each side of with a large and nearly triangular concave on posterior part, narrowly and moderately emarginated in middle and widely roundly emarginated on both sides of posterior margin, the portions between middle and lateral emarginations narrowly rounded at apices. Internal reproductive organ of genitalia (Fig. 4): vagina stout, with diverticulum and spermatheca situated at apex and median oviduct in middle of ventral side; spermatheca sac-shaped and rounded at apex, distinctly expanded apicad and bent in middle, provided with a long and thin accessory gland at base; diverticulum nearly gourd-shaped, global at apical part, with apex bent dorsally; spermatheca and diverticulum surrounded with a strongly sclerotized bracket at base, which is confluent in middle and extending to median oviduct on ventral side, the opening of spermatheca arising from left end of the sclerotized bracket on dorsal side.

Body length: 11.5–15.0 mm; width: 3.0–4.0 mm.

#### Distribution.

China (Hubei, Sichuan, Shaanxi, Gansu).

#### Remarks.

In the original publication (Wittmer 1983), this species was described based on female types collected in Sichuan, China. Later some male characters were supplemented (Wittmer 1995). In the present study, it is redescribed and provided with illustrations of genitalia of both sexes for the first time. And it is newly recorded in Hubei province of China.

## Supplementary Material

XML Treatment for Themus (Themus) dimorphus

XML Treatment for Themus (Themus) testaceicollis
